# Muslim communities learning about second-hand smoke: a pilot cluster randomised controlled trial and cost-effectiveness analysis

**DOI:** 10.1038/npjpcrm.2015.52

**Published:** 2015-08-27

**Authors:** Sarwat Shah, Hannah Ainsworth, Caroline Fairhurst, Helen Tilbrook, Aziz Sheikh, Amanda Amos, Steve Parrott, David Torgerson, Heather Thompson, Rebecca King, Ghazala Mir, Kamran Siddiqi

**Affiliations:** 1 Department of Health Sciences, University of York, York, UK; 2 York Trials Unit, Department of Health Sciences, University of York, York, UK; 3 Centre for Population Health Sciences, The University of Edinburgh, Medical School, Edinburgh, UK; 4 The Office of The Director of Public Health, Leeds City Council, Leeds, UK; 5 Leeds Institute of Health Sciences, University of Leeds, Leeds, UK

## Abstract

**Background:**

In the United Kingdom, men of Bangladeshi and Pakistani origin have higher smoking rates than the general population. This makes non-smokers in their households more vulnerable to second-hand smoke (SHS) exposure than the general population.

**Aims:**

The aim of this study was to investigate the feasibility of implementing and pilot testing the effectiveness and cost-effectiveness of a ‘Smoke-free Homes’ (SFH) intervention in Islamic religious settings to encourage families of Bangladeshi and Pakistani origin to apply smoking restrictions in their homes.

**Methods:**

We allocated Islamic religious settings (clusters) to either receive SFH—an educational intervention—or to a control arm. Within each cluster, we recruited households with at least one smoker and one non-smoker. SHS exposure among non-smokers was measured using salivary cotinine.

**Results:**

Seven (50%) clusters were randomised to each trial arm. A total of 468 households were assessed for eligibility and 62% (*n*=289) were eligible, of which 74% (*n*=213) agreed to participate in the trial. Six of the seven intervention clusters delivered the intervention, and all clusters were retained throughout the trial. In all, 81% (*n*=172) of households provided data at follow-up. No evidence of a difference in log cotinine level was observed (adjusted mean difference −0.02, 95% confidence interval (CI) −1.28–1.23, *P*=0.97) between the two trial arms. The direct mean cost of delivering the intervention was £18.18 per household (range £3.55–42.20).

**Conclusions:**

It was possible to recruit, randomise and retain Islamic religious settings and participant households. However, some of the original assumptions, in particular our ability to collect primary outcome data, need to be revisited before a definitive trial.

## Introduction

Second-hand smoke (SHS) exposure causes serious respiratory and cardiovascular illnesses accounting for up to 1% of the global disease burden.^1^ In recent years, several countries have implemented policies to protect non-smokers from SHS exposure.^2^ In 2007, smoke-free legislation banned smoking in all enclosed public places in England,^3^ which encouraged more smokers to quit^4^ and led to a substantial decline in SHS exposure in public places^2,5^ and improvements in health.^6^ Although the decline in SHS exposure in public and work places was significant,^7^ the impact on smoking restrictions in homes was limited.^8^ Living in a smoke-free home is vital to reducing SHS exposure and its associated harms. This could also encourage smoking cessation.^9^ However, studies report variable effectiveness of the interventions designed to modify smoking behaviour and restrict smoking in homes.^10,11^

SHS exposure is likely to be high among certain marginalised social groups in which smoking is increasingly concentrated, notably lower socio-economic^7,12^ and some minority ethnic groups.^13^ These include communities of Bangladeshi and Pakistani origin, in which rates of smoking among men are higher than those in the general population.^13^ As living with a smoker is an important determinant of SHS exposure,^14,15^ these groups are likely to have higher levels of exposure than the general population. The extent of SHS exposure among minority ethnic groups in the United Kingdom has not been quantified; however, we found that in areas predominantly populated by South-Asian populations, smoking took place regularly in front of children in 42% of households with smokers.^16^ Another study found higher saliva cotinine levels, a measure of recent exposure to cigarette smoke,^17^ among children of Bangladeshi origin than children from other ethnic groups.^18^ The fact that, despite having high levels of motivation, smokers of Bangladeshi and Pakistani origin often fail to modify their smoking behaviours signifies the need for interventions that are tailored to their sociocultural norms.^19^

Promoting health in minority ethnic groups requires an understanding of both ‘surface’ and ‘deep’ dimensions of cultural sensitivities.^20^ The ‘deep’ dimensions, such as religious and sociocultural constructs, help in connecting with the beliefs, values and structures of communities, thereby enhancing salience, acceptability and uptake of health interventions.^21^ Over 90% of people of Bangladeshi and Pakistani origin are Muslims,^22^ and half of them attend mosques at least once a week in the United Kingdom.^23^ There is emerging consensus among Muslims on the religious prohibition of the use of tobacco-containing products.^24^ Several indirect references to Islamic literature are interpreted as a discouragement of tobacco use on the basis of its addictive nature and health hazards.^24^ Religion is an important determinant of beliefs and attitudes towards tobacco use in Bangladeshi- and Pakistani-origin Muslim communities,^25,26^ and it can influence health behaviour. Many believe that tobacco use, even if not explicitly prohibited, is in conflict with Islamic teaching.^25^

No systematic effort has yet been made to study the use of Islamic religious beliefs and settings and the position of faith leaders in influencing smoking behaviours among Muslims. Hence, we developed a Smoke-Free Homes (SFH) intervention to be delivered by Muslim faith leaders in Islamic religious settings to modify their congregations’ smoking behaviour and encourage them to implement smoking restrictions in their homes. In this paper, we present the findings of a pilot trial, designed to assess the feasibility of delivering the effectiveness and cost-effectiveness of this intervention.

## Materials and methods

A detailed trial protocol is published elsewhere.^27^ A summary is presented here.

### Trial design

This was a two-arm pilot cluster randomised controlled trial.

### Setting/clusters

This study was conducted in islamic religious settings (clusters) of Sunni denomination in Birmingham and West Yorkshire, including the following: (1) mosques that host communal prayers, convene study circles for men/women and/or have regular Quran classes for children; (2) Islamic schools (Madrassas) for children; (3) men’s/women’s circles. Those situated within a one-mile radius of another participating cluster were excluded.

### Participants

Attendees of the clusters were approached by the faith leaders and the researchers. Posters, leaflets and announcements after communal prayers were used to promote recruitment. Researchers collected contact details from those expressing interest, assessed their eligibility, obtained consent and completed baseline assessments. Eligible, consenting households in which one or more members attended the respective Islamic religious setting, and which had at least one non-smoker and one smoker, were recruited to the trial.

### Interventions

The clusters were randomised to the intervention and control group in a 1:1 ratio.

#### SFH intervention

The faith leaders in clusters allocated to the intervention arm were trained to deliver the SFH resource to respective audience (Supplementary Material). Designed to encourage change in smoking behaviours, this resource included a guide to group discussions, key take-home messages, role plays, quizzes and games. The activities were tailored to suit different audiences and age groups and delivered in between regular prayers/circle meetings/Quran classes. Take-home leaflets contained key facts about harms associated with SHS and benefits of smoke-free homes. The intervention period lasted for ~3 months. The control clusters did not receive any intervention, but they were offered the SFH resource upon completion of the study.

### Outcomes

We assessed non-smokers’ SHS exposure by measuring salivary cotinine levels using a gas–liquid chromatography technique.^28^ Salivary cotinine concentration is strongly associated with exposure to SHS and has a half-life of 20 h.^29,30^ The feasibility outcome measures included recruitment and attrition rates for clusters and participating households, response rates to the household surveys and collection of saliva samples, the costs associated with delivering SFH, an estimate of the difference in saliva cotinine levels between the two groups and the intra-class correlation coefficient (ICC). Other outcome measures included the proportion of (i) smokers in each cluster; (ii) smokers in each cluster who reported an intention to quit; (iii) households in each cluster that implemented smoking restrictions in their homes.

All data were collected at the time of recruitment and follow-up (5 months post randomisation), which included a household survey completed by an adult.

### Sample size

No formal power calculation was undertaken. A feasible sample size was based on the need to have at least four clusters per arm^31^ to allow for basic statistical analysis. Allowing for attrition, and taking into account the varying sizes of the clusters and their denominations, seven clusters per arm were chosen as a feasible number to recruit. From each cluster, we intended to recruit a maximum of 50 households, resulting in a maximum sample size of 350 households in each arm of the trial.

### Randomisation

Clusters were centrally randomised by the York Trials Unit, in a 1:1 ratio using minimisation.^32^ Minimisation factors included the average size of Friday congregations and the number of consenting households. Randomisation was staggered as clusters were randomised one by one soon after completing household recruitment.

### Statistical methods

All analyses were conducted in STATA v13 (StataCorp)^33^ according to the principles of intention to treat. All statistical tests were two sided at the 5% significance level. Baseline characteristics of the clusters and households were summarised by the trial arm. Continuous measures were reported as means and s.d. and categorical data as counts and percentages.

The threshold to assess SHS exposure through saliva cotinine levels was set at 0.1 ng/ml. Saliva cotinine level of 12 ng/ml or more indicated active smoking.^34^ A comparison was undertaken to investigate the feasibility of studying this measurement and to calculate an estimate for the likely effect size and ICC. Analysis was conducted with the clusters as the unit of analysis and the mean cotinine level in the cluster as the outcome. Saliva cotinine levels were highly positively skewed, and thus values were log transformed and were analysed in a regression model weighted by cluster size, including mean baseline cotinine level and trial arm as covariates. The following were analysed by analysis of covariance weighted by cluster size and adjusting for baseline proportion of the measure and trial arm:

In each cluster at follow-up,

- The proportion of smokers
- The proportion of smokers who reported an intention to quit
- The proportion of households that had smoking restrictions implemented in the home

We conducted field monitoring of the intervention activities. In addition, Islamic religious teachers were trained to record the number and type of activities conducted in the activity checklist provided.

The economic component involved collecting individuals’ use of health-care resources, including any contact with general practitioners and practice nurses (surgery or home visits), prescriptions received, hospital visits and hospital admissions. The number of services used was multiplied by unit costs of care to estimate a cost profile for the period between randomisation and follow-up for individuals in the intervention and control groups. A National Health Services perspective was taken to estimate training costs. Costs were driven by the cost of trainer travel and travel time.

### Ethics statement

Local NRES Committee and University of York granted ethical approval (REC reference: 12/YH/0242).

## Results

### Recruitment

**Figure 1:**
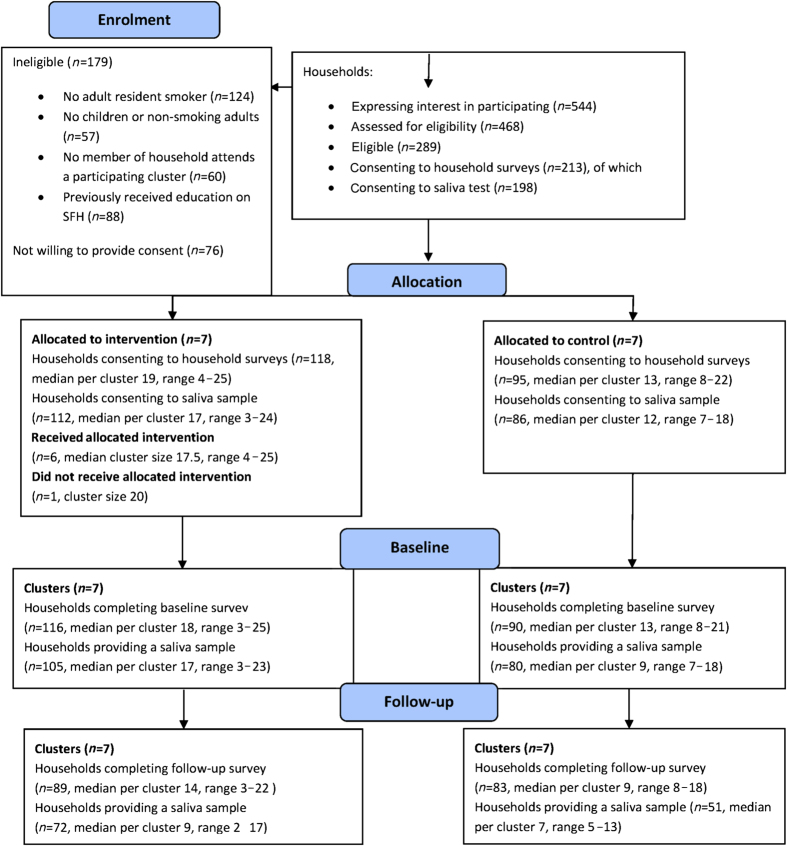
The flow of participants through the trial.

The flow of trial participants is presented in a Consolidated Standards Of Reporting Trials diagram^35^ in Figure 1.

Of the 24 Islamic religious settings approached and assessed for eligibility, 79% (19/24) agreed to participate; however, as per protocol, only 14 were recruited. Eligible consenting households were recruited to the trial between November 2012 and September 2013. A total of 544 households from all 14 participating clusters expressed an interest in taking part in the MCLASS trial, of which 86% (468/544) were assessed for eligibility; 14% (76/544) were not contactable. Among those assessed, 62% (289/468) were eligible, and of those eligible 74% (213/289) agreed to participate in the trial. The most common reason for ineligibility was not having a resident smoker in the household (26%, 124/468). Among the 213 households that consented to participate in the trial, 87% (185/213) provided a cotinine saliva sample at baseline: 90% (105/118) and 84% (80/95) in the intervention arm and control arm, respectively. Household survey questionnaires were received from 97% (206/213), 98% (116/118) and 95% (90/95) in the intervention and control clusters, respectively.

The median length of time between recruitment of first and last household in a cluster was 114 days (range 82–207 days). Table 1 presents location and minimisation factors of participating clusters by allocation. The two trial arms were comparable with respect to average size of the clusters and number of consenting households.

### Baseline

Baseline characteristics of the respondents in participating households were broadly comparable across the intervention and the control arms, except for ethnicity, with a greater proportion of people of Bangladeshi and Pakistani origin (96.7% (87/90)) in the control arm compared with that in the intervention arm (75.4% (86/114)). Respondents were predominantly male (82% (169/206)) and non-smokers (61.2% (126/206)) (Table 2).

Out of 185 saliva samples obtained, 69% (128/185) indicated that the household members were exposed to SHS at baseline (74% (78/105) in the intervention arm and 63% (50/80) in the control arm). About 7.6% (14/185) of samples were indicative of either active smoking or smokeless tobacco use, and 9.2% (17/185) were insufficient to perform the test (Table 3).

### Losses and exclusions

All clusters were retained throughout the trial. Of all recruited households, 81% (172/213) were retained at follow-up: 75% (89/118) of households from the intervention clusters and 87% (83/95) from control clusters. In all, 58% (123/213) provided follow-up samples for salivary cotinine: 64% (72/118) in the intervention arm and 59% (51/95) in the control arm.

### Intervention fidelity

Six out of seven intervention clusters delivered the SHF intervention, with variable fidelity. Religious teachers from some intervention clusters recorded activities on the activity sheet provided; however, most of them did not record any activity.

### Saliva cotinine analysis

Out of 123 saliva samples obtained at follow-up, 66.7% (82/123) were still exposed to SHS: 75% (54/72) in the intervention arm and 54.9% (28/51) in the control arm (Table 4).

No evidence of a difference was observed in the log cotinine level (adjusted mean difference (AMD) −0.02, 95% confidence interval (CI) −1.3 to 1.2, *P*=0.97) between the intervention arm and the control arm. Similarly, in a linear regression weighted by cluster size and adjusting for baseline proportion, no difference was observed between the intervention group and the control group in the (a) proportion of adults smoking (AMD in proportions 2.1, 95% CI −6.4 to 10.7, *P*=0.6); (b) the proportion of smokers who reported an intention to quit (AMD in proportions 16.4, 95% CI −4.5 to 37.3, *P*=0.1); (c) the proportion of households with smoking restrictions (AMD in proportions 0.03%, 95% CI −11.6 to 11.7, *P*=1.0).

The ICC relating to salivary cotinine levels was negligible (<0.00001, 95% CI 0.0–0.1), indicating that the cotinine levels of participants in the same cluster were not strongly related.

The direct cost of delivering the intervention was a mean cost of £18.18 per household (range £3.55 to £42.20). Health service use was completed by trial participants with 1–15% of data missing.

## Discussion

### Main findings

Our study is the first trial in the United Kingdom reporting the use of Islamic religious settings in influencing health-related behaviour of their congregations. Recruitment and retention rates for clusters were encouraging. Of all those who were approached, approximately two-third of clusters were recruited and all were then retained throughout the study period. A large number of households expressed an interest in taking part in the trial, but recruitment still fell substantially short of the initial target. The majority of eligible households consented to complete the household surveys; however, the number of these consenting to providing saliva sample was lower. Similarly, the completion rate for the household survey was higher than that for providing a saliva sample at follow-up. No evidence of a difference in saliva cotinine level between the intervention and control arms was found. The ICC indicated that the cotinine levels of participants in the same cluster were not strongly related. Importantly, the findings of the economic analysis suggest that SFH in these religious settings is a very low-cost intervention to deliver from an National Health Services perspective. Only very modest effectiveness is therefore required to ensure that the intervention is cost-effective.

### Strengths and limitations of this study

The study design had several strengths. The eligibility criteria for clusters were kept to a minimum to ensure good representation of cluster setup (size and range of facilities offered by clusters such as schools, social centres and leisure facilities). Most Islamic religious settings were willing to participate in the study, as reflected by our recruitment rates. This study has helped establish the feasibility of conducting health promotion interventions in an Islamic religious setting, and it acts as a ‘pathfinder’ for future trials using such settings.

Our study met several obstacles. The recruitment of individuals was challenging despite the support received from faith leaders. The barriers we encountered in recruitment, data collection and engagement with mosques need consideration in future trial(s). These are explored in more depth in qualitative data, which will be published separately. Social concerns and attitudes within the communities in which research was conducted seemed to have a key role in the willingness to participate in the study. Our recruitment officers were from the Muslim faith and often from the same ethnic background as the communities they were approaching, which facilitated recruitment. However, we underestimated the time it would take in navigating the different decision-making structures and in building rapport with the ‘gatekeepers’ in various religious settings. Moreover, female recruitment officers faced more difficulties in approaching men in mosques for possible recruitment compared with their male counterparts. Those Islamic religious settings catering for Shia denomination could not be recruited. The generalisability of findings with respect to denomination may therefore be an issue; however, Sunni Muslims comprise ~95% of the British Muslim community.^36^

No formal sample size calculation was performed. However, because we had a negligible ICC, then with the larger number of clusters, the loss of power due to analysis of covariance from losing a degree of freedom was compensated by the predictive value of the covariate.

### Interpretation of findings in relation to previously published work

Some studies report faith-based health interventions to have a positive effect on targeted Muslim ethnic minorities.^37^ However, most of the existing evidence on health programmes taking account of ‘faith dimension’ comes from church settings in the United States in African-American communities.^38^ A faith-based intervention was found to have a substantial increase in physical activity among African-American women in a randomised controlled trial.^39^ The evidence is generally methodologically weak, but it is indicative of potential benefit.^21,38^ Lessons can be drawn from reviewing such literature that may also be applicable to other faith-based communities. Nevertheless, there is a need to develop evidence of this nature for ethnic minorities in the United Kingdom.

### Implications for future research, policy and practice

Despite the above-mentioned challenges, the MCLASS pilot trial findings can help in moving forwards to a definitive trial, as follows. Our cluster recruitment and retention rates reaffirmed our approach of an effective engagement with mosques. This required developing an understanding of the individual mosque administrative structure and internal dynamics that helped us tailor the approach to engaging with the mosque. The type of mosque and its function in the community, i.e., schools, social centres, leisure facilities and so on, had an impact on issues such as women’s attendance and youth participation. This diversity needs to be taken into consideration while defining mosques’ inclusion criteria and the extent to which they could engage with their communities on health-related matters. Our experience of challenges in collecting individual participant data, particularly collecting saliva samples, suggest that in a future trial we need to adopt better strategies to collect data, such as anonymous cross-sectional data in order to address issues around trust and confidentiality.

The study does not objectively report on the fidelity of the intervention, as we did not conduct any direct observations of SFH-related activities using a fidelity index or any exit interviews of the participants. A future study of such interventions should include robust fidelity measures to assess the extent to which an intervention is implemented. We also need to consider how to strengthen the intervention as the point estimates suggest little or no effectiveness. It is imperative to offer more comprehensive training (that includes practical exercises) and support to faith leaders than was offered in this study—e.g., planning about when and how to deliver intervention sessions with faith leaders, or speaking to congregants alongside faith leaders and so on.

### Conclusions

Our study shows that it was possible to recruit, randomise and retain Islamic religious settings and households in the pilot trial. However, some of the original assumptions, in particular our ability to collect primary outcome data, need revisiting before a definitive trial. It is a low-cost intervention. If we can address the limitations outlined to strengthen the intervention, if this proves effective, this is we believe certainly worth doing, particularly in the context of this currently under-served population.

## Figures and Tables

**Table 1:** Location and minimisation factors of participating Islamic religious centres by allocation

*Characteristic*	*Intervention (*n*=7)*	*Control (*n*=7)*	*Total (*n*=14)*
*Location*			
Birmingham	1 (14.3)	2 (28.6)	3 (21.4)
Bradford	2 (28.6)	2 (28.6)	4 (28.6)
Leeds	4 (57.1)	3 (42.9)	7 (50.0)
			
*Average size*			
Small	2 (28.6)	3 (42.9)	5 (35.7)
Medium	2 (28.6)	2 (28.6)	4 (28.6)
Large	3 (42.9)	2 (28.6)	5 (35.7)
			
*Consenting households*			
<10	1 (14.3)	2 (28.6)	3 (21.4)
10–19	3 (42.9)	4 (57.1)	7 (50.0)
20–29	3 (42.9)	1 (14.3)	4 (28.6)

**Table 2:** Lead adult characteristics at baseline

*Lead adult*	*Intervention *n* (%)*	*Control *n* (%)*	*Total *n* (%)*
*Sex, *n* (%)*	N*=116*	N*=90*	N*=206*
Male	96 (82.8)	73 (81.1)	169 (82.0)
Female	20 (17.2)	17 (18.9)	37 (18.0)
			
*Age, years*	N*=115*	N*=88*	N*=203*
Mean (s.d.)	37.1 (13.1)	34.0 (12.3)	35.7 (12.8)
Median (IQR)	35 (28, 42)	33.5 (25, 40)	35 (27, 41)
Min, max)	(16, 74)	(16, 72)	(16, 74)
			
*Smoking status, *n* (%)*	N*=116*	N*=90*	N*=206*
Smokes everyday	39 (33.6)	27 (30.0)	66 (32.0)
Smokes sometimes	7 (6.0)	7 (7.8)	14 (6.8)
Does not smoke	70 (60.3)	56 (62.2)	126 (61.2)
			
*Smoked in the past, *n* (%)*	N*=111*	N*=88*	N*=199*
Yes	61 (55.0)	47 (53.4)	108 (54.3)
No	50 (45.0)	41 (46.6)	91 (45.7)
			
*Ethnicity, *n* (%)*	N*=114*	N*=90*	N*=204*
White	1 (0.9)	1 (1.1)	2 (1.0)
Pakistani	62 (54.4)	64 (71.1)	126 (72.0)
Bangladeshi	24 (21.1)	23 (25.6)	47 (26.9)
ndian	2 (1.8)	0 (0.0)	2 (1.1)
Black/black British	0 (0.0)	1 (1.1)	1 (0.5)
Other^a^	25 (21.9)	1 (1.1)	26 (12.7)
			
*Education continued after age of 16 years, *n* (%)*	N*=114*	N*=89*	N*=203*
Yes	50 (43.9)	52 (58.4)	102 (50.3)
No	64 (56.1)	37 (41.6)	101 (49.8)
			
*Employment, *n *(%)*	N*=113*	N*=90*	N*=203*
In employment or self-employed	63 (55.8)	63 (70.0)	126 (62.1)
Unable to work because of poor health	3 (2.7)	3 (3.3)	6 (3.0)
Look after home/family	10 (8.9)	6 (6.7)	16 (7.9)
Unemployed	10 (8.9)	5 (5.6)	15 (7.4)
Retired	8 (7.1)	1 (1.1)	9 (4.4)
Student	14 (12.4)	10 (11.1)	24 (11.8)
Other	5 (4.4)	2 (2.2)	7 (3.5)

Abbreviation: IQR, interquartile range.

a Includes the following: Afghani, Middle Eastern, Algerian, Sudanese and Syrians.

**Table 3:** Baseline salivary cotinine levels by trial arm

*Baseline cotinine level*	*Intervention (*n*=105)*	*Control (*n*=80)*	*Overall (*n*=185)*
*Classification of saliva sample, *n* (%)*			
Insufficient sample	10 (9.5)	7 (8.8)	17 (9.2)
Not exposed	17 (16.2)	23 (28.8)	40 (21.6)
Exposed (passive smoker)	68 (64.8)	46 (57.5)	114 (61.6)
Possible tobacco user	10 (19.5)	4 (5.0)	14 (7.6)
			
*Supplied by adult* a *, *n* (%)*	N*=66*	N*=47*	N*=113*
Insufficient sample	4 (6.0)	1 (2.1)	5 (4.4)
Not exposed	13 (19.7)	17 (36.2)	30 (26.6)
Exposed (passive smoker)	41 (62.1)	25 (53.2)	66 (58.4)
Possible tobacco user	8 (12.1)	4 (8.5)	12 (10.6)
			
*Supplied by child* a *, *n* (%)*	N*=30*	N*=20*	N*=50*
Insufficient sample	5 (16.7)	3 (15.0)	8 (16.0)
Not exposed	4 (13.3)	3 (15.0)	7 (14.0)
Exposed (passive smoker)	21 (70)	14 (70.0)	35 (70.0)
			
*Exposed samples* b	N*=78*	N*=50*	N*=128*
Mean (s.d.)	18.4 (76.3)	5.8 (19.6)	13.5 (60.9)
Median (IQR)	0.6 (0.3, 2.1)	0.4 (0.2, 0.9)	0.5 (0.2, 1.7)
(Min, max)	(0.1, 588.1)	(0.1, 112.3)	(0.1, 588.1)

Abbreviation: IQR, interquartile range.

a Where data on the provider of the sample was given.

b Summary statistics for the raw salivary cotinine level in the samples with a level ⩾0.1 ng/ml.

**Table 4:** Follow-up salivary cotinine levels by trial arm

*Follow-up cotinine level*	*Intervention (*n*=72)*	*Control (*n*=51)*	*Overall (*n*=123)*
*Classification of saliva sample, *n* (%)*			
Insufficient sample	3 (4.2)	4 (7.8)	7 (5.7)
Not exposed	10 (13.9)	14 (27.5)	24 (19.5)
Passive smoker	54 (75.0)	28 (54.9)	82 (66.7)
Possible tobacco user	5 (6.9)	5 (9.8)	10 (8.1)
			
*Supplied by adult* a *, *n* (%)*	*N=44*	N*=35*	N*=79*
Insufficient sample	1 (2.3)	2 (5.7)	3 (3.8)
Not exposed	7 (15.9)	13 (37.1)	20 (25.3)
Exposed (passive smoker)	31 (70.5)	16 (45.7)	47 (59.5)
Possible tobacco user	5 (11.4)	4 (11.4)	9 (11.4)
			
*Supplied by child* a *, *n *(%)*	N*=22*	N*=14*	N*=36*
Insufficient sample	1 (4.6)	2 (14.3)	3 (8.3)
Not exposed	3 (13.6)	1 (7.1)	4 (11.1)
Exposed (passive smoker)	18 (81.8)	10 (71.4)	28 (77.8)
Possible tobacco user	0 (0.0)	1 (7.1)	1 (2.8)
			
*Exposed samples*^b^	N*=59*	N*=33*	N*=92*
Mean (s.d.)	3.7 (11.5)	4.9 (11.5)	4.1 (11.5)
Median (IQR)	0.4 (0.2, 0.9)	0.4 (0.2, 1.0)	0.4 (0.2, 1.0)
(Min, max)	(0.1, 60.3)	(0.1, 54.4)	(0.1, 60.3)

Abbreviation: IQR, interquartile range.

a Where data on the provider of the sample was given.

b Summary statistics for the raw salivary cotinine level in the samples with a level ⩾0.1 ng/ml.

## Notes

AS is Joint Editor-in-Chief of *npj Primary Care Respiratory Medicine*, but was not involved in the editorial review of, nor the decision to publish, this article. All other authors declare no conflict of interest.
