# Supplementary material for: Muslim communities learning about second-hand smoke: a pilot cluster randomised controlled trial and cost-effectiveness analysis
Source: NPJ Prim Care Respir Med. 2015 Aug 27;25:15052–. doi: 10.1038/npjpcrm.2015.52 (PMC4551097; doi:10.1038/npjpcrm.2015.52)
Supplement: Supplementary Information [file npjpcrm201552-s1.pdf]

Smoke free Homes resource for Muslim religious teachers

http://www.leedsnorthccg.nhs.uk/your-health/staying-healthy/stopping-smoking/resource-pack-for-muslim-teachers/
